# Genetic engineering and heterologous expression of the disorazol biosynthetic gene cluster via Red/ET recombineering

**DOI:** 10.1038/srep21066

**Published:** 2016-02-15

**Authors:** Qiang Tu, Jennifer Herrmann, Shengbiao Hu, Ritesh Raju, Xiaoying Bian, Youming Zhang, Rolf Müller

**Affiliations:** 1 Department of Microbial Natural Products, Helmholtz Institute for Pharmaceutical Research Saarland, Helmholtz Centre for Infection Research and Department of Pharmaceutical Biotechnology, Saarland University, Campus E8.1, 66123 Saarbrücken, Germany; 2 Shandong University – Helmholtz Joint Institute of Biotechnology, State Key Laboratory of Microbial Technology, School of Life Science, Shandong University, Jinan 250100, People’s Republic of China; 3 College of Life Science, Key Laboratory of Microbial Molecular Biology of Hunan Province, Hunan Normal University, Changsha 410081, Hunan Province, People’s Republic of China

## Abstract

Disorazol, a macrocyclic polykitide produced by the myxobacterium *Sorangium cellulosum* So ce12 and it is reported to have potential cytotoxic activity towards several cancer cell lines, including multi-drug resistant cells. The disorazol biosynthetic gene cluster (*dis*) from *Sorangium cellulosum* (So ce12) was identified by transposon mutagenesis and cloned in a bacterial artificial chromosome (BAC) library. The 58-kb *dis* core gene cluster was reconstituted from BACs via Red/ET recombineering and expressed in *Myxococcus xanthus* DK1622. For the first time ever, a myxobacterial *trans*-AT polyketide synthase has been expressed heterologously in this study. Expression in *M. xanthus* allowed us to optimize the yield of several biosynthetic products using promoter engineering. The insertion of an artificial synthetic promoter upstream of the *disD* gene encoding a discrete acyl transferase (AT), together with an oxidoreductase (Or), resulted in 7-fold increase in disorazol production. The successful reconstitution and expression of the genetic sequences encoding for these promising cytotoxic compounds will allow combinatorial biosynthesis to generate novel disorazol derivatives for further bioactivity evaluation.

Natural products from microorganisms, fungi, plants and insects display a broad spectrum of biological activities. Currently, approximately 49% of anti-infectives compounds and 61% of anticancer pharmaceutical agents in clinical use are natural products or their derivatives^1^. Over the last decades, myxobacteria have become well known producer organisms, offering a rich and valuable source of natural products^2^,^3^. Most of these compounds are biosynthesized by multifunctional megasynthetases, such as polyketide synthases (PKSs)^4^, nonribosomal peptide synthetases (NRPSs)^5^ and hybrids thereof 6. Genes encoding these PKSs and NRPSs in bacteria are often clustered together on the chromosome, so a gene cluster can be cloned into a vector and then transferred to a heterologous host for functional expression^7^. Recent studies demonstrate the usefulness of heterologously expressed secondary metabolite pathways for the production of natural products^8^. Heterologous expression can improve fermentation yields and generate new natural or synthetic products that can be evaluated as potential pharmacological agents in the course of targeted derivatization or structure-activity relationship studies^9^.

Reconstructing biosynthetic gene clusters in various vectors for heterologous expression in more productive hosts can help show how newly discovered biosynthetic gene clusters function. Derivatives of the new available pharmacologically active compounds can then be produced by biomolecular re-engineering and combinatorial biosynthesis^10^.

Large clusters of genes that encode the enzymes for natural product biosynthesis have been difficult to engineer using conventional technology. Red/ET recombineering in combination with TAR (Transformation-associated recombination) cloning is necessary for large biosynthetic gene clusters to be engineered conveniently^11^,^12^.

Red/ET recombineering is based on *in vivo* homologous recombination in *E. coli*^13^,^14^,^15^. The greatest advantage of the technology is that it works regardless of restriction enzyme binding sites or the size of DNA fragments to be shuffled. This technology has made it much easier to genetically manipulate complex biosynthetic pathways in bacteria. Over the past decade, several complete biosynthetic pathways from fastidious bacteria have been heterologously expressed via Red/ET recombineering, e.g. myxochromide S and myxothiazol in *Pseudomonas putida* and *M. xanthus*^16^,^17^,^18^,^19^, epothilones in *M. xanthus*^19^, human alpha-defensin 5 mature peptide in *Pichia pastoris*^20^, nikkomycin in *Streptomyces ansochromogenes*^21^, pretubulysin in *P. putida* and *M. xanthus*^22^, luminmycin and glidobactin in *E. coli* Nissle1917^23^,^24^, salinomycin in *Streptomyces coelicolor*^25^ and even a minimal set of genes for magnetosome biosynthesis from the magnetotactic bacterium in *Rhodospirillum rubrum*^26^.

Secondary metabolite gene clusters in microbes express natural products with potential medicinal and values agricultural qualities^27^. However, many of the microorganisms hosting these biosynthetic pathways grow slowly even in optimized laboratory conditions and can not be genetically manipulated^2^. Heterologous expression of complete secondary metabolite pathways plays a significant role in hunting for new natural products and developing them into useful drugs^16^. Many heterologous expression instruments for secondary metabolite pathways have been reported so far, ranging from targeted expression by shuttle vectors to the random expression of large DNA fragments from chromosomes by transposition^9^.

Disorazols, a family of structually complex macrocyclic polyketides, are produced by the myxobacterium *Sorangium cellulosum* So ce12 (Fig. S1) and firstly isolated in 1994^28^. Disorazols inhibit cancer cell proliferation at low picomolar concentrations by preventing tubulin polymerization and inducing destabilization of microtubules, which ultimately leads to the induction of apoptosis^29^,^30^,^31^. The extraordinary potency of disorazols fostered their development as peptide-conjugates for cancer therapy^32^,^33^ and encouraged the generation of new and simplified disorazol derivatives by means of chemical synthesis^34^,^35^,^36^. However, there are no reports to date on genetic engineering approaches for the production of new analogs of the disorazol compound class.

The *dis* biosynthetic gene cluster was identified by transposon mutagenesis. In 2005, the cluster was cloned into a BAC or cosmid library of *S. cellulosum* So ce12 by two independent groups^37^,^38^. The clusters showed the anticipated *disA-C* genes encoding hybrid *trans*-AT type I PKS/NRPS megaenzymes, and also another gene, *disD*, that encoded an additional acyl transferase protein (Fig. 1).

According to the biosynthetic model, seven malonyl-CoA units and one serine are incorporated as extender units, forming half of the disorazol bis-lactone core unit. Two polyketide monomers may dimerize to form disorazol via the thioesterase (TE) domain^38^, possibly requiring an esterase encoded by *orf3*′ 37 (Table S4).

The native strain produces only small amounts of disorazols (~1 mg per liter fermentation medium) and is difficult to cultivate^28^. Consequently, it is challenging to produce large quantities of disorazol for further development. Using an amenable heterologous host should be a rational way to assure higher and stable disorazol yields and possibly optimize its structure by molecular engineering.

Here we report the Red/ET recombineering of the *dis* biosynthetic gene cluster into a stable vector containing a p15A replication origin and a MycoMar transposase element. When the *dis* gene cluster was transposed into the chromosome of the heterologous host *M. xanthus* DK1622 several disorazol derivatives were produced. Subsequent gene deletions proved that only the *disA-D* genes and not *orf9* or the putative esterase gene *orf3*′ were needed for disorazol production^37^. Further, we also improved disorazol production in the heterologous host *M. xanthus* DK1622 by replacing the native promoter of the *disD* gene encoding a discrete AT protein with an artificial synthetic promoter.

## Results and Discussion

### Reconstitution of the disorazol A biosynthetic gene cluster

The disorazol A biosynthetic gene cluster has been cloned, sequenced and identified previously from a BAC library of So ce12^37^. The BAC contained most of the *dis* gene cluster from *disA* to *disD*. However, the BAC pBeloBAC11-dis was a large and low copy vector and very difficult to transfer between hosts for heterologous expression. To construct a more efficient expression vector and to insert elements for transfer and expression into different heterologous hosts, we sequentially modified the original BAC (pBeloBAC11-dis) by Red/ET recombineering^13^,^14^. The backbone of pBeloBAC11-dis was replaced by a cassette containing the p15A replication origin (*p15A ori*), the origin of transfer (*oriT*) for conjugation purposes, two inverted repeats (IRs), a MycoMar transposase gene (*Tps*) for transposition, an inducible promoter *tetR-*P_tet_ for driving the *dis* gene cluster upstream of *disA* and a kanamycin resistance gene for selection in *M. xanthus* DK1622.

**Figure 2:**
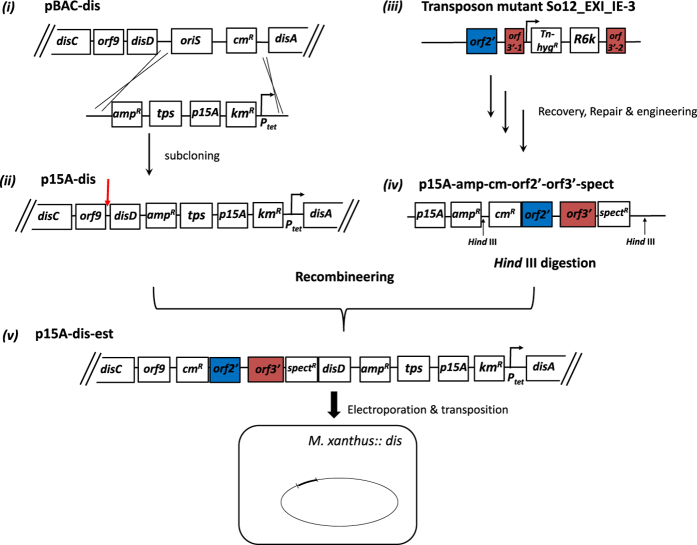
Diagram of disorazol A gene cluster engineering. Firstly, the backbone of plasmid pBeloBAC11-dis (i) was replaced by p15A ori-tps casstte to form p15A-dis (ii) which containing an original MycoMar transposon by Red/ET recombineering. In this way *dis* gene cluster was driven by P_tet_ promoter. Then, the interrupted esterase gene *orf3’* in pTn-Rec_IE2 plasmid (iii) from transposon mutant So12_EXI_IE-3^36^ was recovered, repaired and engineered to form the vector p15A-amp-cm-orf2’-orf3’-spect (iv) that contained the whole length of the esterase gene *orf3’*. Next, linear DNA fragment released by *Hind* III was integrated into disorazol vector p15A-dis (ii) to get the final construct p15A-dis-est (v) via Red/ET recombinantion. Finally, two types of modified vectors p15A-dis (ii) and p15A-dis-est (v) were electroporated into *M. xanthus* respectively and kanamycin-resistant colonies were selected for further analysis. *Hind* III restriction sites used for releasing linear fraction “*cm^R^-orf2’-orf3’-spect^R^*” were indicated in ↑. The insertion site of the linear fragment DNA containing orf2’ and orf3’ gene was marked with ↓.

In the resulting construct p15A-dis, the *dis* gene cluster (containing *disA-D* and *orf9*) is in a relatively high copy number vector (20–30 copies per cell in *E. coli*). Instead of the native promoter, expression in this vector is controlled by a tetracycline inducible promoter in this vector works in several heterologous hosts, e.g. *E. coli, M. xanthus* and *P. putida*^39^ (Figs 2 and S2).

We previously found that disorazol production was no longer detectable when an esterase gene (*orf3*′) was mutated by transposon insertion in mutant strain So12_EXI_IE-3^37^. This mutated esterase gene was implicated in bis-lactone formation during disorazol biosynthesis. We recovered plasmid pTn-Rec_IE2 (Fig. S6), which contained several genes near the transposition in the mutant So12_EXI_IE-3. The transposon was found in the middle of the carboxyl esterase gene *orf3*′ (only 6.7 kb upstream of the *disA* start codon).

The plasmid pTn-Rec_IE2 also included a S-adenosyl methionine (SAM) dependent methyl transferase gene *orf2*′^37^. As the product of *orf2*′ may *O*-methylate the OH group at C-6′ adjacent to the *orf3*′ gene, it might also be essential for disorazol biosynthesis (Fig. S6, Table S4). Hence, we inserted both, the repaired carboxyl esterase gene *orf3*′ and the SAM-dependent methyl transferase gene *orf2*′ together into p15A-dis to form p15A-dis-est by Red/ET recombineering. To gain the fusion plasmid p15A-dis-est, firstly, two separate PCR cassette “*cm*^*R*^” and “*spect*^*R*^” with suitable homologous arms to the region (containing two *Hind III* restriction sites in both sides) between the *orf9* and the *disD* genes were introduced into the vector, respectively. After digestion by Hind III restriction enzyme in correct clones, the linear fragment “*cm*^*R*^*-orf2*′*-orf3*′*-spect*^*R*^” was integrated to obtain the final construct p15A-dis-est. By this, the *cm*^*R*^ gene was introduced to drive *orf2*′ and *orf3*′ genes. Likewise, the *spect*^*R*^ gene was introduced to drive the *disD* gene (Figs 2 and S2).

Certain gene products may be toxic to the host cell, potentially limiting the nature of downstream applications when introduced into *E. coli* directly at high copy number^40^. All *E. coli* strains containing the *dis* gene cluster with the native promoter were found to carry mutations after recombineering. Therefore, it was very challenging to obtain the expression construct containing the *dis* gene cluster directly in *E. coli* because the growth of the host was impeded. We reasoned that one of the *dis* proteins interfered with a primary metabolic pathway in *E. coli* to disrupt growth. To address this issue, an inducible promoter P_tet_, was used to regulate gene expression. P_tet_ is a versatile tetracycline-based regulatory system that is usually used to selectively control expression of downstream genes^39^. No other promoter system is suitable for so many diverse hosts, including *E. coli, M. xanthus* and *P. putida*^10^,^22^,^23^,^41^. Besides, P_tet_ had already enabled several mixed PKS/NRPS natural products to be produced in heterologous hosts unrelated to the native producing organisms, such as myxochromide S from myxobacterium *Stigmatella aurantiaca*, which has been engineered into *P. putida*^19^.

The transposon method, which was also applied in this study, is clearly more stable and efficient than using shuttle vectors^19^. Several indispensable elements were inserted into the target vectors, for instance *Tps* and *oriT*. The mariner transposon MycoMar is frequently used in Gram-negative hosts for genetic modification^42^,^43^ and to transfer and integrate a gene cluster into the chromosome of heterologous host strains^19^,^22^. The transformation efficiency of large gene sets is higher when using the MycoMar transposon than using homologous recombination, as has been described for the heterologous expression of epothilone and myxochromide S^19^. This powerful tool for transforming large genes was used in the disorazol heterologous expression system to make it easier to integrate the *dis* gene cluster into the genome of host strains. The *oriT* was also incorporated for conjugation in other heterologous hosts strains, such as *P. putida*^44^.

### Heterologous expression of *dis* gene cluster in *M. xanthus* DK1622

**Figure 3:**
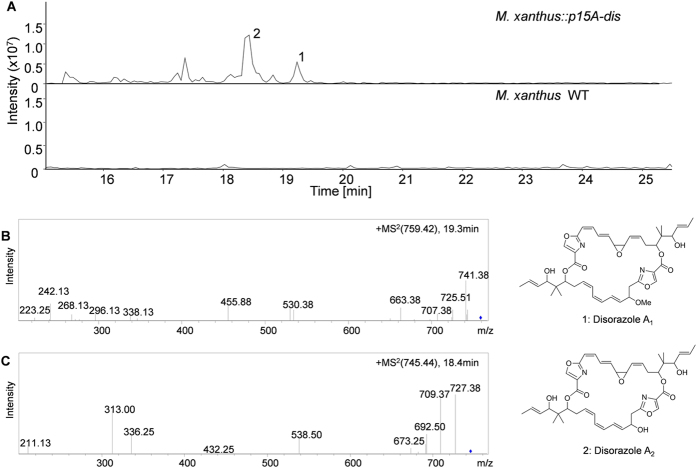
Analysis of disorazol production in *M. xanthus* wildtype (WT) and *M. xanthus::p15A-dis* grown at 30 °C and induced with 0.5 μg/ml AHT. (**A**) HPLC-MS analysis (base peak chromatogram [BPC] *m/z* 720–780) of *M. xanthus::p15A-dis* and *M. xanthus* WT. (**B**) MS^2^ fragmentation pattern of disorazol A_1_ (**1**). (**C**) MS^2^ fragmentation pattern of disorazol A_2_ (**2**).

Both expression constructs p15A-dis and p15A-dis-est (Fig. S2) were introduced into the heterologous host *M. xanthus* DK1622 by electroporation as previously described^19^. The *dis* gene cluster was randomly transposed into the chromosome of *M. xanthus*. Transformants were screened on CTT agar containing kanamycin to select for *M. xanthus::p15A-dis* and *M. xanthus::p15A-dis-est* mutants. Six randomly chosen colonies of each mutant were verified by PCR^19^, which confirmed that the *dis* gene cluster had been integrated into the *M. xanthus* chromosome in each case. All the checked mutants contained the whole disorazol gene clusters. Several resulting mutants *M. xanthus::p15A-dis* and *M. xanthus::p15A-dis-est* were cultivated (both induction by anhydrotetracycline (AHT)) for compound extraction and detection. All the mutants produced detectable amounts of disorazols by the analysis of high performance liquid chromatography-tandem mass spectrometry (HPLC-MS)^45^. We have found small amounts of various disorazol compounds (including disrazols A_1_, A_2_, A_3_, A_4_, B_2_, B_4_ and F_2_) in both extracts of *M. xanthus:: p15A-dis* and *M. xanthus:: p15A-dis-est* (Figs 3 and S3, Table S2), upon comparing the secondary metabolite profiles from *M. xanthus* wild type strain and mutants. As expected, these results indicate that the chosen set of genes is sufficient to produce the polyketide-nonribosomal peptide skeleton of the disorazols.

Unexpectedly, without the *orf2*′ and the *orf3*′ genes, *M. xanthus:: p15A-dis* can also produce disorazols. The overall yields of disorazols in *M. xanthus:: p15A-dis* (averagely were 0.4 mg/L) match with that in *M. xanthus:: p15A-dis-est* (averagely were 0.42 mg/L). Result exhibited that the *orf3*′ gene is dispensable in the disorazol biosynthesis in the chosen heterologous host. There might be an enzyme that can substitute for the similar function of the *orf3*′ gene product in *M. xanthus* host. The *dis* gene cluster could be inactivated in the transposon mutant So12_EXI_IE-3 due to a strong polar effect^37^ because it is adjacent to the *disA* gene, possibly preventing downstream genes in an operon from being transcribed^46^,^47^.

The HPLC-MS and NMR data showed that the major compound in both mutants *M. xanthus:: p15A-dis* and *M. xanthus:: p15A-dis-est* was disorazol A_2_ which constituted 55% of final product after purification from crude extracts (Figs S3 and S4, Table S3), whereas disorazol A_1_ was 20%. But in the native host So ce12, disorazol A_1_ was the chief component (nearly 70% after purification, 10 times higher than disorazol A_2_) produced among the 29 derivatives^28^. The most probable explanation was that an *O*-methyl transferase that methylates the OH group at C-6′ was absent in the heterologous expression of *dis* gene cluster. This methyl transferase gene could be possibly located elsewhere in the chromosome of the native producer So ce12, which still needs further investigation. Only small amounts of the C-6′ methylated disorazols A_1_, A_3_ and A_4_ were produced in *M. xanthus* (Fig. S3), which might be due to partial methylation by a nonspecific *M. xanthus O*-methyl transferase. After 5 L fermentation of mutant strain *M. xanthus::p15A-dis*, the yield of disorazol A_2_ was approximately 0.24 mg/L, which is 5-fold higher than described in the native producer strain So ce12^28^,^48^. The result unambiguously demonstrated again that secondary metabolites can be produced in heterologous hosts under the control of the versatile P_tet_ promoter which encouraged further investigation of disorazol formation.

### Biological activity of disorazol compounds

After having isolated disorazols from our heterologous host *M. xanthus*, biological studies revealed exceptional high cytotoxicity of disorazol A_2_ on eukaryotic cells. We determined IC_50_ values against several established human cancer cell lines from different origin and disorazol A_2_ strongly inhibited the growth of these cell lines with IC_50_ values between 0.05 and 4.9 nM (Table 1). However, compared to disorazol A_1_, the antiproliferative activity of disorazol A_2_ was less pronounced on most cell lines, except for human U-937 histiocytic lymphoma. Most likely, the higher IC_50_ values for disorazol A_2_ are due to the lack of a methyl group at C-6′ compared to disorazol A_1_, which in turn might lead to a less favourable binding to the traget structure tubulin. Nevertheless, when compared to other anticancer drugs, such as epothilone B or vinblastine, disorazol A_2_ is still much more effective *in vitro*^29^,^49^.

### Optimized production with biomolecular technology

An unusual feature of the disorazol biosynthetic gene cluster is that it has only one discrete AT domain on the DisD module, and hence it is called a *trans*-AT type of PKS^50^. In recent years, *trans*-AT PKSs have been found in an important group of biosynthetic enzymes that produce bioactive natural products, including pederin, rhizoxin, leinamycin, myxovirescin, chivosazol and psymberin^51^,^52^. Accessing functionally-optimized polyketides by modifying PKSs through targeted synthase re-engineering is an encouraging approach to optimize natural products for application^52^. However, in contrast to ATs from *cis*-AT PKSs, the mechanisms and structures of *trans*-acting ATs are still unexplored.

The *disD* gene has been modified here to show how *trans*-acting ATs affect the disorazol biosynthesis pathway. In order to enhance the expression of the solitary AT domain, we introduced another strong promoter P_cp25_ upstream of the *disD* gene. P_cp25_ is a highly active, constitutive lactococcal consensus promoter, whose sequence has already been reported^53^,^54^. Previous studies have illustrated that overexpression of single genes or multigene transcriptional units by promoter exchange in myxobacteria can improve the production of secondary metabolites^19^,^55^,^56^,^57^.

On the other hand, the role of *orf9* gene (showing similarity to hypothetical proteins), which separates the *disC* and *disD* genes, in the *dis* gene cluster has not been defined^37^. To discover the actual function of the *orf9* gene in disorazol biosynthesis, we inactivated it on the expression construct p15A-dis and then performed heterologous production in *M. xanthus*.

**Figure 4:**
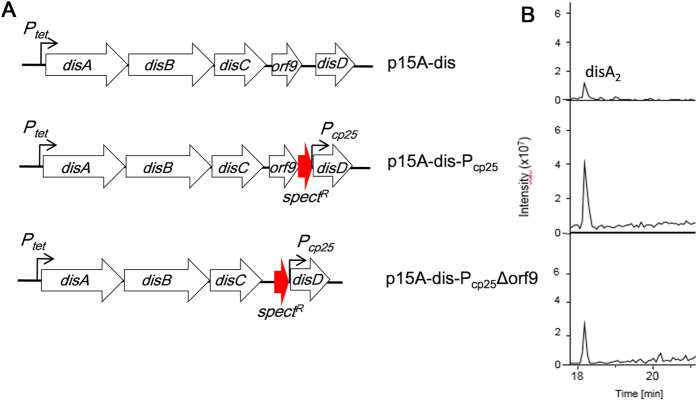
Promoter insertion in front of *disD* gene. (**A**) Three different types of expression constructs used for disorazol heterologous production. The first one is the original plasmid p15A-dis. The second one was modified via Red/ET recombineering by insertion of promoter P_cp25_ in front of *disD* directly. The third one was deletion *orf9* gene by P_cp25-_*spect*^*R*^ so that P_cp25_ was also upstream *disD*. (**B**) Quantification of heterologous disorazol production by HPLC-MS analysis of the culture extracts from different *M. xanthus* DK1622 mutant strains. Sections of extracted ion chromatograms at m/z = 745.45 corresponding to the [M + H]^+^ ion of disorazol A_2_ are illustrated as representative readout of productivity. The *M. xanthus* DK1622 host strains contain one of the three expression constructs shown in (**A**).

The PCR cassette “*P*_*cp25*_*-spect*^*R*^” (P18–P20 in Table S1), containing promoter P_cp25_ and a spectinomycin resistance gene (*spect*^*R*^), with two different pairs of homologous arms, was inserted into p15A-dis by Red/ET recombineering to form two plasmids p15A-dis-P_cp25_ and p15A-dis-P_cp25_Δorf9 (Figs 4A and S5). In the first plasmid p15A-dis-P_cp25,_ the promoter P_cp25_ was inserted directly upstream of the *disD* gene. In the second plasmid, the *orf9* gene was deleted by using a synthetic promoter cassette with selection for spectinomycin resistance to obtain p15A-dis-P_cp25_Δorf9. The *disD* gene was thereby controlled by the P_cp25_ promoter in both expression constructs. The recombinants were analyzed after growth on low-salt Luria-Bertani (LB) broth plates plus spectinomycin. The verified constructs were transformed into *M. xanthus* DK1622 and three randomly picked positive transformants of each type of strain were cultivated to analyze the production by HPLC-MS. To clearly identify disorazol, retention times (RT) and the MS^2^ fragmentation pattern were compared to authentic reference substances. The concentration of disorazol A_2_ in the culture was determined by UPLC-HRMS. A standard curve between peak area and concentration was established from serial dilutions for disorazol A_2_ down to 0.01μg/mL. The peak area of disorazol A_2_ (base peak chromatograms, BPC + 759.3 ± 0.1, RT = 18.2 min) was calculated by BrukerDaltonics compass data analysis 4.0. The yields of all disorazols were estimated from their relative peak areas in the HPLC-MS chromatogram by comparison with the standard curve for each derivative.

**Figure 5:**
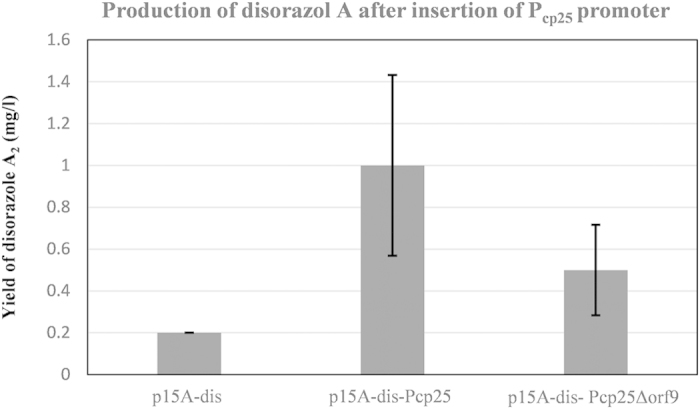
Production of disorazol A after insertion of P_cp25_ promoter. Quantification of disorazol heterologous production by HPLC-MS analysis of the culture extracts from different *M. xanthus* DK1622 mutant strains. All LC-MS- derived area values are normalized to the crude extracts of each sample by method of standard curves. The depicted values are mean values from three independent mutants. Error bars show calculated SDs, yield, control strain.

All the resulting host strains still produced disorazols with growing production titres based on HPLC-MS analysis. The generated *M. xanthus*:: p15A-dis-Pcp_25_ expression host produced on average seven times more disorazol A_2_ compared to *M. xanthus*:: p15A-dis and mutant strain *M. xanthus*:: p15A-dis-P_cp25_Δorf9 produced approximately 2.5-fold when compared to *M. xanthus*:: p15A-dis (Figs 4B and 5). Hence, the *orf9* gene ablation did reduce disorazol production although it was described as having “no functional prediction” in BLAST analysis^37^. The *orf9* gene, following the TE domain, might affect the biosynthetic formation of the final product by incorporation and cyclization of two sides of the disorazol bis-lactone. The successful enhancement of disorazol heterologous production suggested that re-engineering *trans*-AT PKSs domains on the molecular level was a feasible and practicable approach in investigating the characteristic enzymes.

*Trans*-AT PKSs are an important but still less known family of biosynthetic systems in comparison to *cis*-AT PKSs^58^,^59^. There are significant differences in the existing biosynthetic protocols between *trans*-AT and *cis*-AT PKSs. A single discrete AT DisD recognize and load all molonyl-CoAs for all the *dis* PKS modules. Here we change the native promoter of *disD* gene with a stronger and artifical synthetic P_cp25_ promoter which it would increase the transcription of *disD* gene and most likely raises the amount of DisD protein. Sufficient ATs could provide abundant substrates, thus promote the PKS module efficiency for polyketide chain extension of disorazol biosynthesis leading to improved production^52^,^60^,^61^. As *trans*-AT PKSs are a special group of enzymes responsible for natural product biosynthesis in the organisms, it is essential to understand their functions in order to develop more heterologous expression systems for these special polyketides. Here, we have established a pioneer protocol to overexpress an independent AT resulted in increased yield of the final product, which can be used for the production optimzation of *trans*-AT directed natural products in the native or heterologous hosts.

## Methods

### Bacterial strains and culture conditions

All recombineering was performed in *E. coli* strain GB2005 and its derivatives cultured in LB medium and antibiotics (kanamycin [km], 15 μg/ml; ampicillin [amp], 100 μg/ml; spectinomycin [spect], 40 μg/ml; chloramphenicol [cm], 30 μg/ml and tetracycline [tet], 5 μg/ml). The strains used were: GB2005, derived from DH10B by deletion of *fhuA, ybcC* and *recET*^19^,^62^; GB05-red, derived from GB2005 by insertion of the P_BAD_-*gbaA* cassette at the *ybcC* locus^41^,^62^; GB05-dir, derived from GB2005 by the P_BAD_-ETgA operon, which was integrated into the *ybcC* locus in GB2005^41^. The integration ablates expression of *ybcC*, which encoded a putative exonuclease similar to that encoded by Redα. The heterologous host for PKS/NRPS gene cluster expression was *M. xanthus* DK1622 grown at 30 °C in CTT medium (1% casitone, 8mM MgSO_4_, 10mM Tris-HCl, pH 7.6, and 1mM potassium phosphate, pH 7.6)^63^ with or without km (50 μg/ml) before or after introduction of the disorazol gene cluster.

### Red/ET recombineering

All methods were essentially as described previously^62^. By using Red/ET recombineering, 0.3 μg of one linear DNA fragment (either a PCR product or a fragment obtained from restriction enzyme digestion) was electroporated into 50 μl Red/ET-competent *E. coli* cells (such as GB-red cells). After electroporation, colonies grew on the LB-agar plates under selection for the antibiotic resistance gene and then were examined for the intended Red/ET recombination product by restriction analysis with a set of different suitable enzymes.

All PCR reactions carried out using Taq polymerase (Invitrogen GmbH, Karlsruhe, Germany) according to the manufacturer’s protocol. For the amplification of the ~1000 bp cassette with high GC content, DMSO was added to a final concentration of 3%. The conditions using an Eppendorf master cycler were as follows: 10 min at 95 °C to activate the polymerase, denaturation at 95 °C (30 s), annealing at 58 °C (30 s), and extension at 72 °C (35 s); 35 cycles. The PCR product was directly used without any purification.

### Reconstitution of *dis* gene cluster

To harvest the full length of the esterase gene *orf3*′ in plasmid pTn-Rec_IE2, we first changed the backbone of pTn-Rec_IE2 into p15A-amp-orf2′-Tn-hyg in order to get more stable and higher copies of DNA (P3, P4 in Table S1). Then two linear fragments, p15A-amp-orf2′-Tn-hyg digested by *Sal* I and primer P5 (see Table S1), were co-transformed into *E. coli* GB05-dir cells^41^ to remove *R6k-Tn-hyg*^*R*^ genes and recover the whole size of *orf3*′ gene. Thus, we obtained plasmid p15A-amp-*orf3*′ harboring the full-length esterase gene. In order to insert the whole length *orf2*′ and *orf3*′ genes into the disorazol plasmid, we inserted two single PCR cassettes “*spect*^*R*^” and “*cm*^*R*^” with suitable homologous arms (P6-P9 Table S1) into the vector to engineer plasmid p15A-amp-cm-orf2′-orf3′-spect and then digested the new construct with *Hind* III to release the linear cassette “*cm*^*R*^*-orf2*′*-orf3*′*-spect*^*R*^” whose *Hind* III restriction site were homologous to p15A-dis vector. In the last step, the “*cm*^*R*^*-orf2*′*-orf3*′*-spect*^*R*^” cassette was transformed into strain GB-red::p15A-dis strain to generate the final plasmid (Figure S7). Two expression constructs p15A-dis and p15A-dis-est were obtained, containing four core-large genes from the disorazol A pathway (ten PKSs and one NRPS, ~58kb), with the P_tet_ promoter located upstream of the first PKS domain (Fig. 2).

### Electroporation of *M. xanthus* DK 1622

The engineered gene clusters were introduced into the chromosome of *M. xanthus* DK1622 by electroporation. Briefly, *M. xanthus* cells from 1.7 ml of overnight culture with OD600 ~ 0.6 were collected and electrocompetent cells were prepared after washing twice with ice-cold water. A mixture of 50 μl cell suspension in cold water and 3 μg DNA was electroporated (Electroporator 2500, Eppendorf AG, Hamburg, Germany) at 1300V using a 0.1 cm cuvette. After electroporation, the cells were resuspended in 1.7 ml fresh CTT medium, and incubated at 30 °C in a 2 ml Eppendorf tube with a hole punched in the lid on a Thermomixer (Eppendorf) at 11000 r.p.m. for 6 h. Then 1 ml 1.5% CTT agar solution at 42 °C was added to the tube and the cells were plated in soft agar for selection on CTT agar plates supplemented with km (50 μg/ml). Km-resistant colonies appeared after 4 days and were checked by colony PCR as follows. Part of a single colony was washed once in 1 ml H_2_O and resuspended in 100 μl H_2_O. Then, 2 μl of the resulting suspension was used as a PCR template using Taq polymerase according to the manufacturer’s protocol. The disorazol-specific primers used to check the integration of the *disC* gene into the *M. xanthus* chromosome were the same as used in a previous study^16^. For PCR amplification, primers 10 and 11 were used (see Table S1).

### Expression and analysis of disorazol production

Plasmids harboring a core-region or reconstituted dis gene cluster were introduced into *M. xanthus* DK1622 by electroporation. The resulting mutants (*M. xanthus* DK1622:: p15A-dis) were cultivated in 100ml shake flasks containing 30 ml CTT medium. The medium was inoculated with 0.5 mL of the overnight culture and incubated at 30 °C on a rotary shaker at 180 rpm. After induction (anhydrotetracycline, final concentration 0.5 μg/mL) and addition of XAD adsorber resin (2%, 24 h), incubation was continued for 2 more days. The cells and the resin were harvested by centrifugation and extracted with methanol. The extracts were evaporated and then redissolved in 1 mL MeOH. A 5 μL solution was analyzed by HPLC-MS and analysis was performed on an Agilent 1100 series solvent delivery system that was equipped with a photodiode array detector and coupled to a Burker HCTultra ion trap mass spectrometer. Chromatographic conditions were as follows: Luna RP-C_18_ column, 100 × 2 mm, 2.5 μm particle size, and precolumn C_18_, 8 × 3 mm, 5 μm. Solvent gradient (with solvents A [water and 0.1% formic acid] and B [CH_3_CN and 0.1% formic acid]): 20% B from 0 to 20 min, 20% B-95% B within 10 min, followed by 5 min with 95% B at a flow rate of 0.4 mL/min. Detection was carried out in positive ion mode, auto MS^n^. Disorazols were identified by comparison to the retention times and the MS^2^ data of disorazols identified from the original producer in our myxo-database (target screening, Table S2)^28^. The relative production of disorazols was calculated from the peak areas of the extracted ion chromatograms (EICs) of each derivative.

High-resolution mass spectrometry was performed on an Accela UPLC-system (Thermo-Fisher) coupled to a linear trap-FT-Orbitrap combination (LTQ-Orbitrap), operating in positive ionization mode. Separation was achieved on a Waters BEH RP- C_18_ column (50 × 2.1 mm; 1.7 μm particle diameter; flow rate 0.6 mL/min, Waters), with a mobile phase of H_2_O/CH_3_CN (each containing 0.1% formic acid) and a gradient of 5–95% CH_3_CN over 9 mins. UV and MS detection were performed simultaneously. Coupling of HPLC to MS was supported by an Advion Triversa Nanomate nano-ESI system attached to a Thermo Fisher Orbitrap. Mass spectra were acquired in centroid mode at 200–2000 *m/z* with a resolution of R = 30000.

### Target screening method

The HPLC-HR-MS data of crude extracts were further analyzed to identify the known compounds present in the extracts using the software Target Analysis (Bruker Daltonik GmbH). The known compounds were identified on the basis of their high resolution mass, isotope pattern and retention time according to the known method^45^. With this approach, re-isolation of known but less interesting compounds could be avoided whereas unknown compounds with potential bioactivity could be identified easily.

### Isolation of disorazol A_2_

*M. xanthus* containing p15A-dis was cultivated in 5 L CTT medium supplemented with 30 μg/mL kanamycin and 2% XAD 16 resin (after 2 days of incubation) at 30 °C for 5 days^63^. The resin was collected by sieving, washed with H_2_O twice, and then extracted stepwise with acetoacetate (5 L). The extract was concentrated *in vacuo*, followed by suspension in MeOH and extraction with *n*-hexane to defat. The resulting MeOH extract (0.87 g) was fractionated initially on a Sephadex LH-20 column (100 × 2.5 cm) using MeOH as a mobile phase, and 55 fractions were obtained. Fractions containing disorazol A_2_ were subjected to semi-preparative reversed-phase HPLC system (Jupiter Proteo C_12_, 250 × 10 mm, 4 μm, DAD at 254 nm) with an isocratic system of 75% MeOH/H_2_O with 0.05% TFA to yield (1.2 mg, t_R_ ≈ 22 min).

### NMR

NMR spectra were recorded in CD_3_OD on a DRx 500 MHz spectrometer (^1^H at 500 MHz, ^13^C at 125 MHz) equipped with a 5-mm probe and a Bruker Ascend 700 MHz spectrometer (^1^H at 700 MHz, ^13^C at 175 MHz) equipped with a 5-mm TXI cryoprobe system (Bruker Biospin GmbH, Germany). Chemical shift values of ^1^H- and ^13^C-NMR spectra are reported in ppm relative to the residual solvent signal given as an internal standard. Multiplicities are described using the following abbreviations: s = singlet, d = doublet, t = triplet, q = quartet, m = multiplet, b = broad; corrected coupling constants are reported in Hz.

## Additional Information

**How to cite this article**: Tu, Q. *et al.* Genetic engineering and heterologous expression of the disorazol biosynthetic gene cluster via Red/ET recombineering. *Sci. Rep.* **6**, 21066; doi: 10.1038/srep21066 (2016).

## Supplementary Material

Supplementary Information

## Figures and Tables

**Figure 1:**
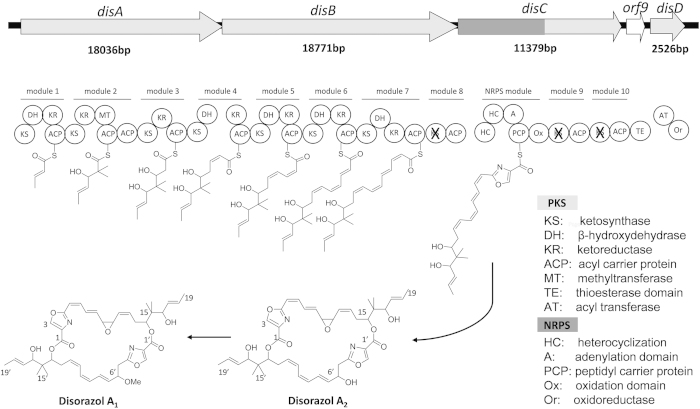
Domain organization of the *dis* biosynthetic gene cluster from *S. cellulosum* So ce12 and a model for biosynthesis of disorazol A_1_ (scheme according to Kopp *et al.*).

**Table 1:** Activity of disorazol A_1_ and disorazol A_2_ against human cancer cell lines.

Human Cell line	Origin	IC_50_ [nM]	
		Disorazol A_1_	Disorazol A_2_
A-431	*epidermoid carcinoma*	1.866	4.908
A-549	*lung carcinoma*	0.072	0.408
HCT-116	*colon carcinoma*	0.032	0.071
HepG2	*hepatocellular carcinoma*	0.002	0.051
HL-60	*acutemyeloid leukemia*	0.058	0.084
K-562	*chronicmyeloid leukemia*	0.074	0.140
KB-3.1	*cervix carcinoma*	0.025	0.106
SW480	*colonadeno carcinoma*	0.030	0.128
U-2 OS	*osteosarcoma*	0.038	0.206
U-87 MG	*glioblastoma-astrocytoma*	0.072	0.119
U-937	*histiocytic lymphoma*	0.293	0.210

IC_50_ values refer to antiproliferative activities.
