# Supplementary material for: Genetic engineering and heterologous expression of the disorazol biosynthetic gene cluster via Red/ET recombineering
Source: Sci Rep. 2016 Feb 15;6:21066. doi: 10.1038/srep21066 (PMC4753468; doi:10.1038/srep21066)
Supplement: Supplementary Information [file srep21066-s1.pdf]

Supplemental Information

Genetic Engineering and Heterologous Expression of the Disorazol Biosynthetic Gene Cluster via Red/ET Recombineering

Qiang Tu, Jennifer Herrmann, Shengbiao Hu, Ritesh Raju, Xiaoying Bian, Youming Zhang and Rolf Müller

Inventory of Supplemental Information

Supplemental data

Figure S1. Chemical structures of disorazols.

Structures of all disorazol derivatives mentioned in this paper. On Page 3.

Figure S2. related to Figure 2. Heterologous expression constructs of two type expression plasmids p15A-dis and p15A-dis-est. On Page 9.

Figure S3. related to Figure 3. HPLC-MS analysis of target screening of extracts from *M. xanthus:: p15A-dis.* On Page 10.

Figure S4. related to Figure 3. ^{1}H NMR spectrum data of disorazol A_2. On Page 11.

Figure S5. related to Figure 5. Modify *disD* gene through Red/ET recombineering. On Page 13.

Figure S6. Construct of the recovered plasmid pTn-Rec_IE2. On Page 7.

Table S1 Oligonucleotides used in this study.

Table S2 related to Figure 3. Target screening analysis data of extracts from *M. xanthus ::p15A-dis.* On Page 10.

Table S3 related to Figure 3. NMR data for disA_2 comparison with the natural product. On Page 11.

Table S4 Proteins encoded on the recovered plasmid pTn-Rec_IE-2 and their putative function in disorazol biosynthesis. On Page 5.

Supplemental experimental procedures

Supplemental references

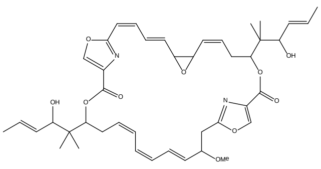
1
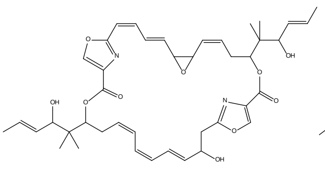
2
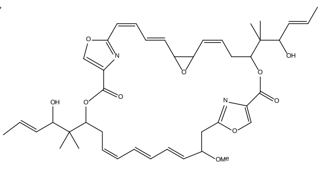
3
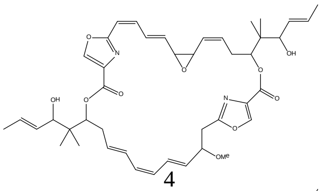
4
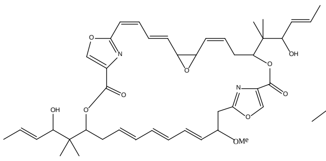
5
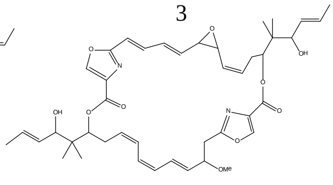
6
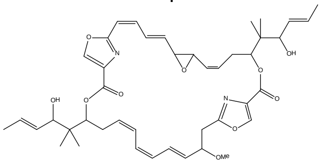
7
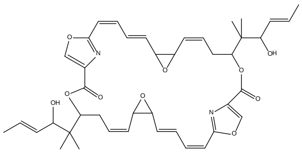
8
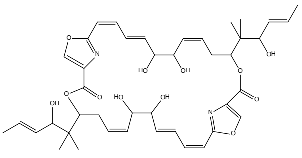
9
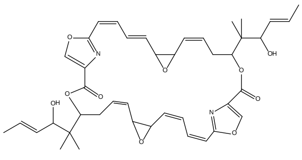
10
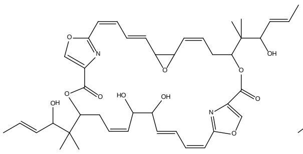
11
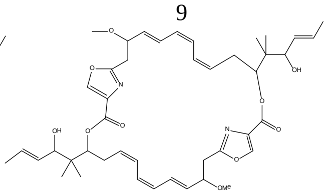
12
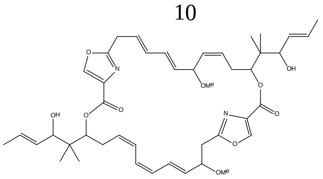
13
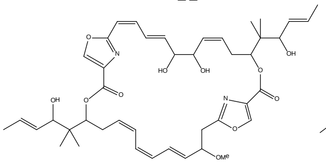
14
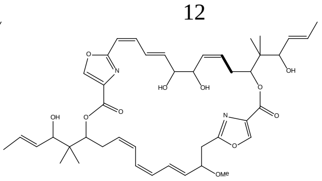
15
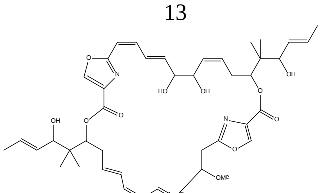
16
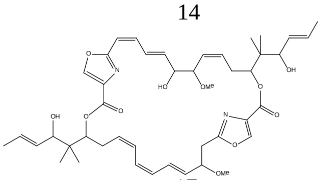
17
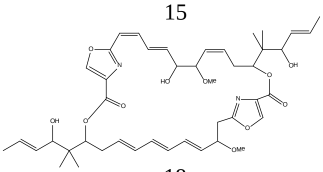
18
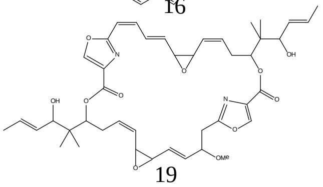
19
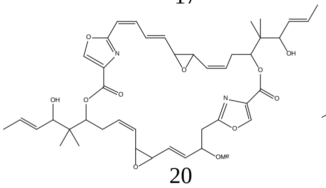
20
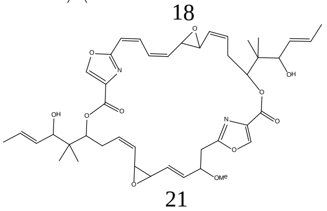
21
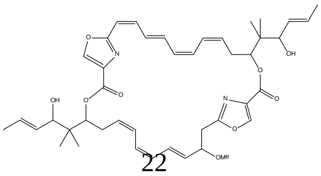
22
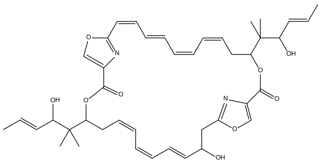
23
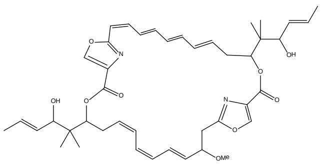
24
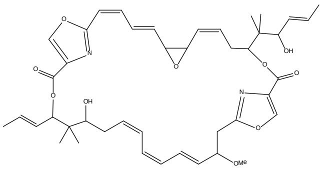
25
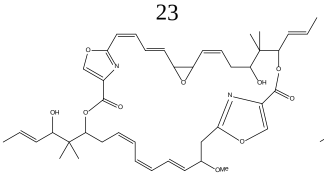
26
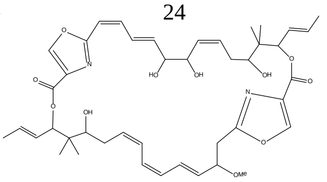
27
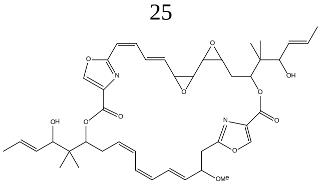
28
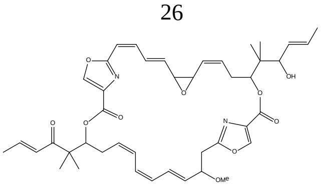
29

Figure S1. Chemical structures of disorazols.

1. Disorazol A_1
2. Disorazol A_2
3. Disorazol A_3
4. Disorazol A_4
5. Disorazol A_5
6. Disorazol A_6
7. Disorazol A_7
8. Disorazol B_1
9. Disorazol B_2
10. Disorazol B_3
11. Disorazol B_4
12. Disorazol C_1
13. Disorazol C_2
14. Disorazol D_1
15. Disorazol D_2
16. Disorazol D_3
17. Disorazol D_4
18. Disorazol D_5
19. Disorazol E_1
20. Disorazol E_2
21. Disorazol E_3
22. Disorazol F_1
23. Disorazol F_2
24. Disorazol F_3
25. Disorazol G_1
26. Disorazol G_2
27. Disorazol G_3
28. Disorazol H
29. Disorazol I

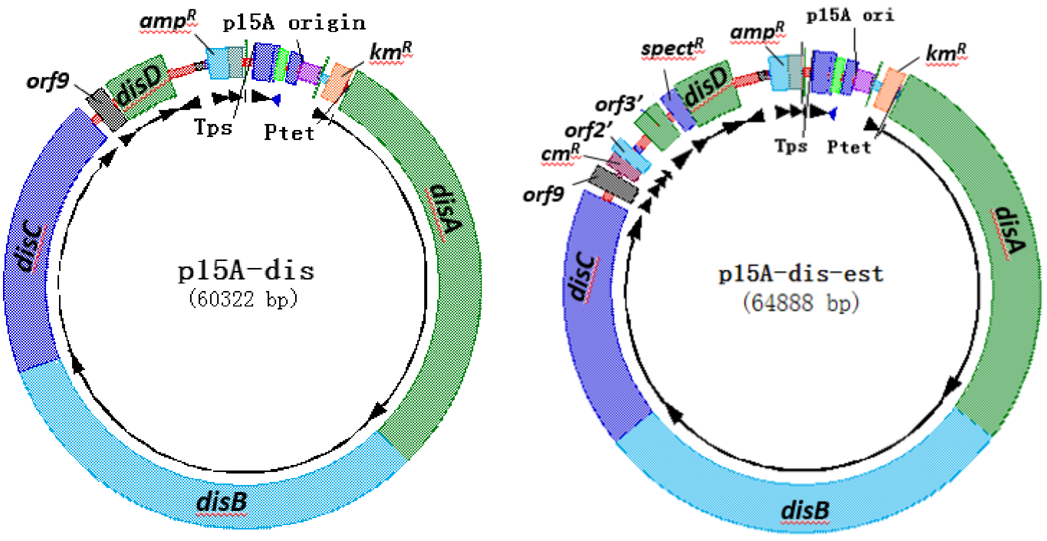

1 **2**

Figure S2. Heterologous expression constructs of two type of expression plasmids p15A-dis and p15A-dis-est.

Construct 1 is p15A-dis. Then we insert the repaired carboxyl esterase gene *orf3'* and the SAM-dependent methyl transferase gene *orf2'* together into p15A-dis by Red/ET recombineering to form construct 2 p15A-dis-est. After insertion, two genes are in the middle of *orf9* gene and *disD* gene.

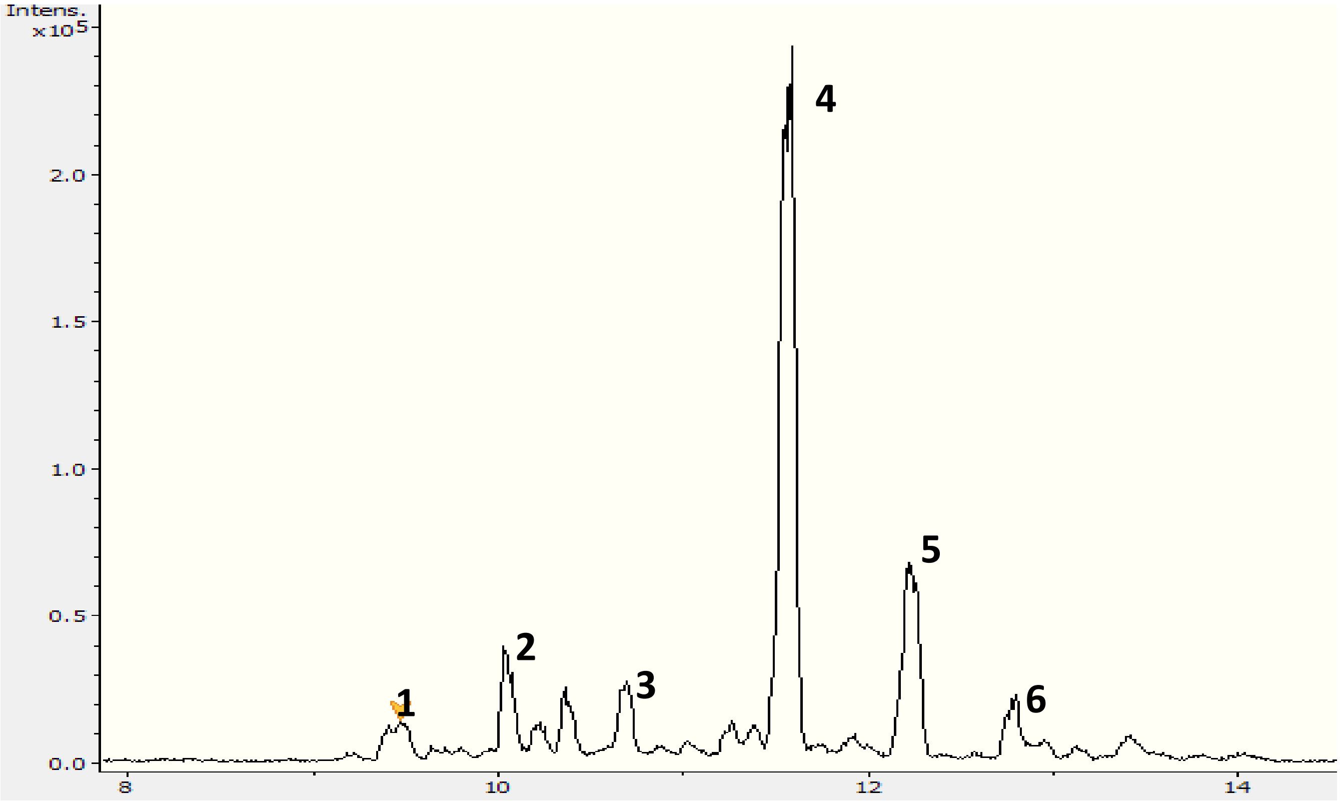

Figure S3. related to Figure 3. HPLC-MS analysis (BPC *m/z* 720-780) of extracts from *M. xanthus:: p15A-dis.*

The peaks are $disB_4$ (1), *m/z* 761 $[M+H]^+$; dis762 (2), *m/z* 763 $[M+H]^+$; $disB_2$ (3), *m/z* 779 $[M+H]^+$; $disA_2$ (4), *m/z* 745 $[M+H]^+$; $disA_1$ (5), *m/z* 759 $[M+H]^+$; $disF_2$ (6), *m/z* 729 $[M+H]^+$.

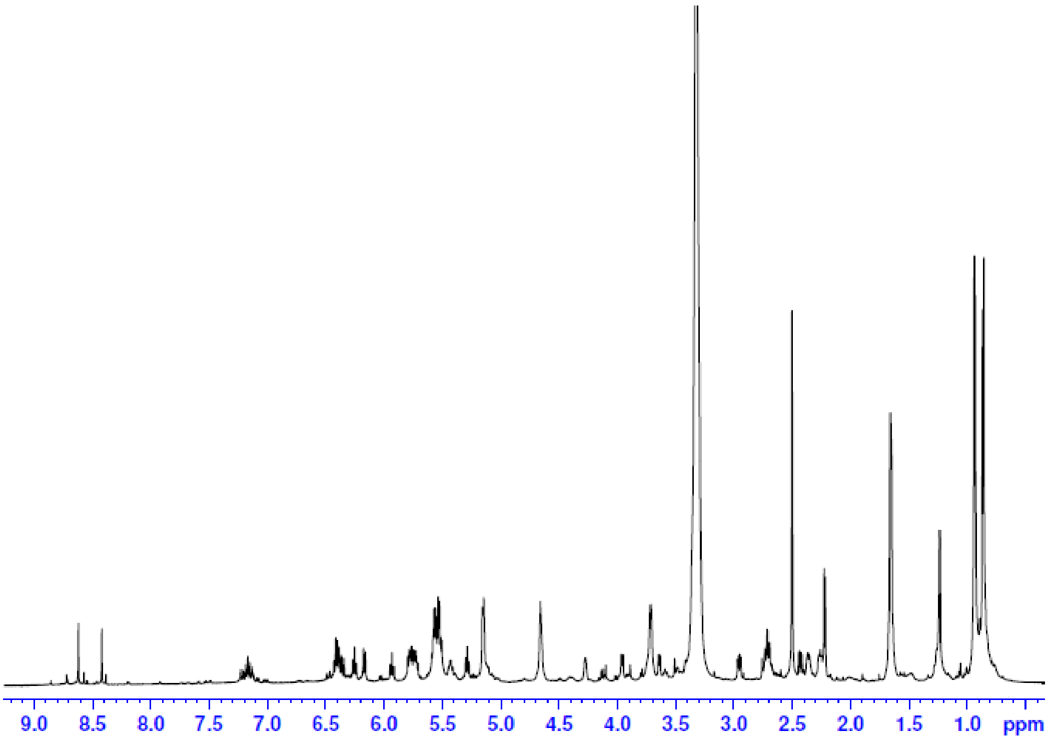

Figure S4. ^{1}H NMR spectrum of disorazol A_2.

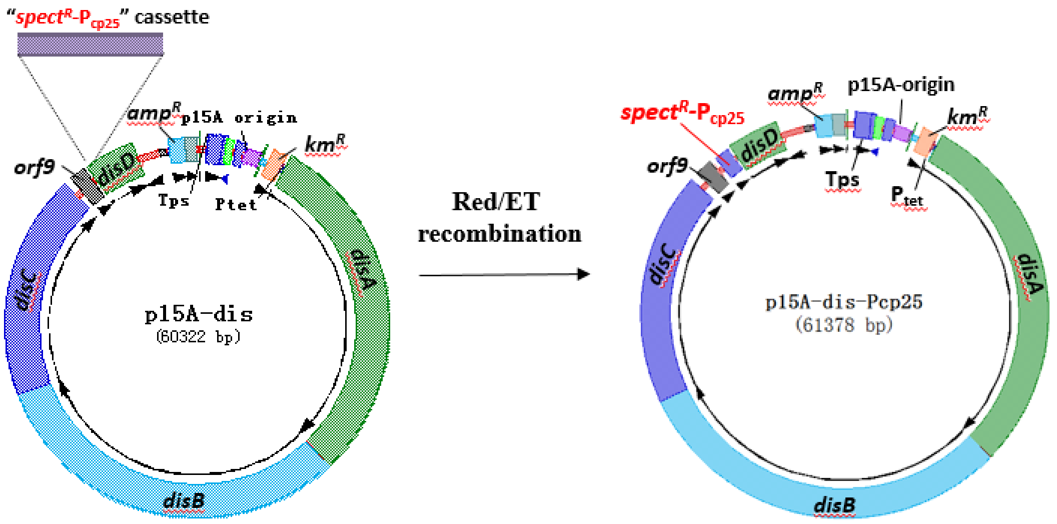

1

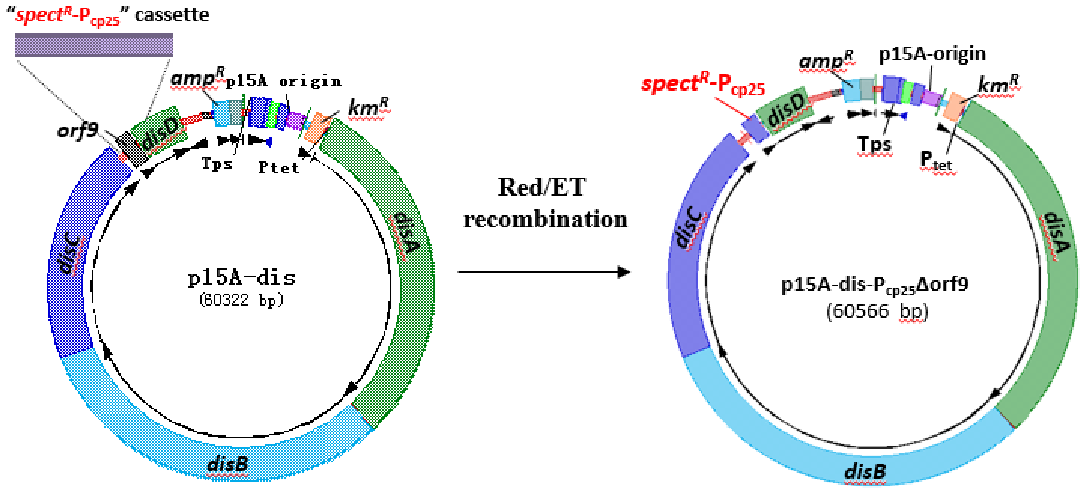

2

Figure S5. Modify *disD* gene through Red/ET recombineering.

1: only insert promoter P_{cp25} in front of *disD* gene.

2: insert promoter P_{cp25} in front of *disD* gene by deletion *orf9* gene.

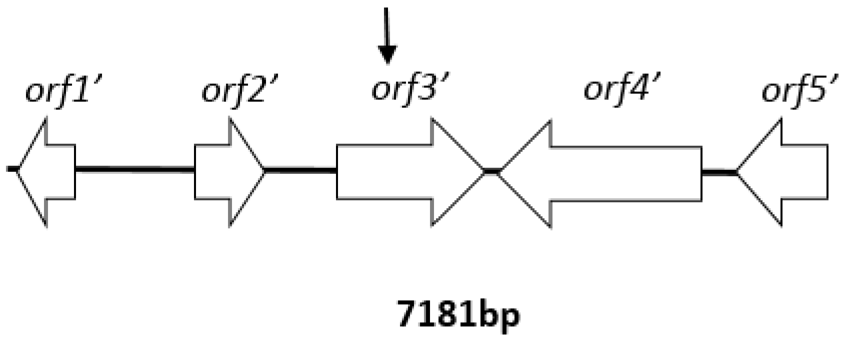

Figure S6. Construct of the recovered plasmid pTn-Rec_IE2. (data according to Kopp et al.)

Diagram of the genes encoded adjacent to the transposition site of mutant So12_EXI_IE-3 that was cloned into the recovered plasmid pTn-Rec_IE-2. ↓ is the location of the transposon insertion site.

Table S1 Oligonucleotides used in this study

name	sequences(5'-3')	Notes
P1	AATGTCGCACAATGTGCGCCATTTTTCACTTCACAGGTCAACGCTCACGCTGACGCTGTCCGCCGAGACCGACTTGGGCTTCTACCTGCGGATCCTGGTGATGATGGCGGGATCGTTG	Used to replace the backbone of disBAC plasmid
P2	AATGAATAGTTCGACAAAAATCTAGATAACGAGGATCAACGATGGAGCAGGACGCCATTGCGATCATCGGCGTAGCGTGCCGATTTCCCGGGATCCTGTGACGGAAGATCACTTCG	
P3	TCCGCTGCATCCTGGATCTGGATATGGACAGGAAGGCGTTTGAAGAAGTCGAGTTTGAACAAAACAACTTATATCG	Used to replace the backbone of pTn-Rec_IE2 plasmid
P4	CTCAGCCCGCGTCGAGCGCGTAGACGGCCGTGGCCGTCTTTCGCCCCGTCAGGTCTTACCAATGCTTAATCAGTGAG	
P5	TACCGTCGCGGCCACGCGCATCTGGGGCCCCCTCGGCATGAACGATACGATGTACAACCCGCCCGCGGAGCTCCACGAGCGCGTGGCGGCCACGGGCTACTCGACC	Used to delete R6k-Tn-hyg genes in order to form full length of esterase gene
P6	CAGATCGCTGAGATAGGTGCCTCACTGATTAAGCATTGGTAAGCTTGAGACCCTTGCCCGGGAAACATGTATGCTTTCATCGGTTCTTCCTCTTATAATTTTTTTAATCTGTTA	Used to form the homology arm to p15A-dis plasmid
P7	CCTCAGCCCGCGTCGAGCGCGTAGACGGCCGTGGCCGTCTTTCGCCTAACGTGACTGGCAAGAGATAT	
P8	TGACCTCCGGCTCGAGCACGCGCGCCAGCGACATGCTCGCATCCTTCTCCGTTACGCCCCGCCCTGCCACTC	Used to form the homology arm to p15A-dis plasmid
P9	TCAGCCCCATACGATATAAGTTGTTTTGTTCAAACTCGACTTCAAGCTTGCGCCGAGGCGAGCCCCTGGCGGGCACGTCGTGGCGGCGCGCCTCCTGGTGTCCCTGTTGATACC	
P10	TGGAAAGCGCGATGACCATCCAGGAGTTTGCCAACTTGTCTGCGGAG	Used to verify *M. xanthus::p15A-dis* mutant.
P11	TGTGGGAGGCGAGGGCCTCGGCGAAGAGGGTGAGGAGCAGGGCCGTCGG	

P12	TGGAGGTCCGCCCGATCGCCGAGGGC<br>GAGCTGCACGAGCGCCTCGCGCGGCA<br>GGAGCCCTTACGCCCCGCCCTGCCACT<br>CATCGCAG	Used to inactivate *disA* gene
P13	TGATAGAGAAAAGTGAAATGAATAGT<br>TCGACAAAAATCTAGGAGGATGATGA<br>CGCCAACTTTTGGCGAAAATGAGAC	
P14	CGTAGGACACCCGGTTGGCGATCGAC<br>GAGTAATCGGCGCTCGCGATCACGGG<br>GTTTCCCTTGTGGAGCTCGTCCTGTTA<br>CGCCCCGCCCTGCCACTCATCGCA	Used to inactivate *disB* gene
P15	TGCGCCGCTCGGCTATTACCAATCGAC<br>CTGGACCAGAAGCGCGCTTTGAACGT<br>CGGGGTAACCTCCCAACTTTTGGCGA<br>AAATGAGAC	
P16	CGGCTCGGTGAGCGAGAGCCGCAGCT<br>CGAAGAAGGGCCACTGATCCAGGGGG<br>AATACCCTTTACGCCCCGCCCTGCCAC<br>TCATCGCAG	Used to inactivate *disC* gene
P17	TGGCCGGCGTACCGGGCGAGGAGCTG<br>ACTCGGCTCTACGCCATCCTGCAAGA<br>GGAATGATGACCAACTTTTGGCGAAA<br>ATGAGAC	
P18	TGAGACCCTTGCCCGGGAAACATGTA<br>TGCTTTCATCGGTTCTTCCTCTCTATCA<br>CTGATAGGGAGTGGTAAAATAACTCT<br>ATCAATGATAGAGTGTCAACAGTACT<br>ATGTGATTATACC	Used to insert P_{cp25} promoter in front of *disD* gene
P19	CGCGCCGAGGCGAGCCCCTGGCGGGC<br>ACGTCGTGGCGGCGCGCCTCCTCCCCG<br>CGAGCACGTGTTGACAATTAATC	
P20	TGCGTTTGATATCGAGCGATCCGCATG<br>ATAGACGACCCCGCGCTGAACCGAGC<br>ACGTGTTGACAATTAATC	Used to delete *orf9* gene by insertion of P_{cp25} promoter in front of *disD* gene

Table S2 related to Figure 3. Target screening analysis data of extracts from *M. xanthus::p15A-dis.*

RT [min]	m/z	Compound	sum formula
4.36	335.16205	Ar001-23-2	$C_{14}H_{27}N_2O_5S_1$
12.48	384.28846	AS_DK1622_383-1	$C_{25}H_{38}N_1O_2$
12.48	384.28846	AS_DK1622_383-2	$C_{25}H_{38}N_1O_2$
5.1	631.27543	Cittilin A	$C_{34}H_{39}N_4O_8$
**12.81**	**729.37322**	**Disorazol F2**	$\mathbf{C_{42}H_{52}N_2O_9}$
**11.58**	**745.36879**	**Disorazol A2**	$\mathbf{C_{42}H_{53}N_2O_{10}}$
**12.24**	**759.38345**	**Disorazol A1**	$\mathbf{C_{43}H_{55}N_2O_{10}}$
**12.24**	**759.38345**	**Disorazol A3**	$\mathbf{C_{43}H_{55}N_2O_{10}}$
**12.24**	**759.38345**	**Disorazol A4**	$\mathbf{C_{43}H_{55}N_2O_{10}}$
**7.77**	**779.37324**	**Disorazol B2**	$\mathbf{C_{42}H_{55}N_2O_{12}}$
**9.49**	**761.36162**	**Disorazol B4**	$\mathbf{C_{42}H_{53}N_2O_{11}}$
**10.04**	**763.37894**	**Disorazol 762**	$\mathbf{C_{42}H_{54}N_2O_{11}}$
7.44	519.25799	DKxanthen-518	$C_{29}H_{35}N_4O_5$
7.8	519.25783	DKxanthen-518	$C_{29}H_{35}N_4O_5$
7.18	535.25311	DKxanthen-534	$C_{29}H_{35}N_4O_6$
7.54	535.25281	DKxanthen-534	$C_{29}H_{35}N_4O_6$
8.07	549.26825	Dkxanthen-548	$C_{30}H_{37}N_4O_6$
3.56	183.09128	Marinoquinoline A	C_{12} H 11 N 2
5.36	225.13794	Marinoquinoline B	C_{15} H 17 N 2
5.84	259.12181	Marinoquinoline C	$C_{18}H_{15}N_2$
2.55	197.12852	Mediacompound-Amb-001	$C_{10}H_{17}N_2O_2$
3.56	245.12887	Mediacompound-Amb-002	$C_{14}H_{17}N_2O_2$
4.01	245.12862	Mediacompound-Amb-003	$C_{14}H_{17}N_2O_2$
3.51	211.14406	Mediacompound-Amb-004	$C_{11}H_{19}N_2O_2$
13.01	416.31467	Myxalamid A	$C_{26}H_{42}N_1O_3$
12.59	402.2989	Myxalamid B	$C_{25}H_{40}N_1O_3$
11.84	388.28299	Myxalamid C	$C_{24}H_{38}N_1O_3$
13.83	430.33012	Myxalamid-430	$C_{27}H_{44}N_1O_3$
5.4	405.16444	Myxochelin A	$C_{20}H_{25}N_2O_7$
3.93	404.18101	Myxochelin B	$C_{20}H_{26}N_3O_6$
13	624.4456	Myxovirescin A	$C_{35}H_{62}N_1O_8$
12.73	622.42858	Myxovirescin B	$C_{35}H_{60}N_1O_8$
16.86	632.44647	Myxovirescin C	$C_{35}H_{63}N_1Na_1O_7$
11.03	642.45472	Myxovirescin Variante KP641	$C_{35}H_{64}N_1O_9$
2.3	261.12324	Tyr-Pro Dioxopiperazin	$C_{14}H_{17}N_2O_3$
2.41	261.1234	Tyr-Pro Dioxopiperazin	$C_{14}H_{17}N_2O_3$

The shadow part are disorazol and its derivatives (in bold in the table).

Table S3 related to Figure 3. NMR data for disA_2 comparison with the natural product.

pos	δ_H, mult (*J* in Hz) Disorazol[1] A_2	δ_H, mult (*J* in Hz) Disorazol[2] A_2
3-H	8.34, s br	8.42, s
5-H	6.18, d br (*11.7*)	6.17, d (*11.9*)
6-H	6.47, dd (*11.7*, *11.9*)	6.41, m
7-H	7.36, dd (*11.9*, *15*)	7.16, dd (*11.4*, *14.7*)
8-H	5.71, m	5.75, m
9-H	3.66, dd (*4.2*, *9.9*)	3.64, dd (*4.4*, *9.9*)
10-H	4.06, dd (*4.2*, *9.7*)	3.96, dd (*4.2*, *10.0*)
11-H	5.34, dd (*9.7*, *11.5*)	5.28, dd (*9.0*, *11.2*)
12-H	5.86, ddd (*5.5*, *11*, *11*)	5.77, m
13-Ha	2.88, m	2.71, m
13-Hb	2.46, ddbr	2.43, dd (*3.2*, *14.6*)
14-H	5.35, dd (*2.5*, *11*)	5.15, m
16-H	3.88, d (*7.5*)	3.73
17-H	5.64, m	5.74, m
18-H	5.71, m	5.15, m
19-H_3	1.74, d (*1*, *6*)	1.65
20-H_3	1.07[a], s	0.94[a], s
21-H_3	1.03[a], s	0.93[a], s
3’-H	8.49, s	8.62, s
5’-Ha	3.11, dd (*5.4*, *14.9*)	2.95, dd (*6.0*, *14.7*)
5’-Hb	2.65, dd (*3.8*, *14.9*)	2.43, dd (*3.3*, *14.4*)
6’-H	4.42, m	4.66, m
7’-H	5.88, dd (*9*, *15.1*)	5.93, dd (*9.4*, *12.0*)
8’-H	6.43, dd (*11*, *15.1*)	6.39, m
9’-H	6.01, dd (*11*, *11*)	6.17, d (*11*)
10’-H	6.37, dd(*11*, *11*)	6.36, m
11’-H	6.48, dd(*11*, *11*)	6.40, m
12’-H	5.55, ddd (*5.5*, *11*, *11*)	5.54, m
13’-Ha	2.88, m	2.72, m
13’-Hb	2.36, m	2.26, m
14’-H	5.35, dd (*2.5*, *11.6*)	5.15, m
16’-H	3.88, d (*7.5*)	3.72, m
17’-H	5.64, m	5.54, m
18’-H	5.71, m	5.50, m
19’-H_3	1.74, d	1.65
20’-H_3	1.02[b], s	0.85[b], s
21’-H_3	1.01[b], s	0.87[b], s

[1]NMR data taken in MeOH-d_4, [2] DMSO-d_6 [a,b] overlapping signals.

Table S4 Proteins encoded on the recovered plasmid pTn-Rec_IE-2 and their putative function in disorazol biosynthesis (data according to Kopp et al.)

Gene	Size (bp)	Proposed function of the similar protein	Similarity/ Identity
orf1'	522	arylesterase-related protein	29%/ 43%
orf2'	591	SAM- dependent methyl transferase	48%/ 58%
orf3'	1284	putative esterase β-lactamase	35%/ 51%
orf4'	1782	adenylate cyclase	31%/ 51%
orf5'	854	outer membrane protein (incomplete)	36%/ 46%

Genetic inactivation of the disorazol biosynthetic genes

As mentioned earlier, ten PKS modules and one NRPS module are encoded in the genes *disA-C* in the conserved disorazol biosynthetic gene cluster. To confirm that *disA-C* is involved in the biosynthesis of disorazol, the module was inactivated by disrupting the gene, including the PKS modules 1 and 5 and the NRPS module, on the p15A-dis expression construct (Figure 1). A 1100 bp fragment conferring chloramphenicol resistance (cm) to the linker region between these modules was separately inserted into the p15A-dis construct by homologous recombination in *E. coli*. The modified deletion constructs pDisA, pDisB and pDisC were screened using low-salt LB plates plus chloramphenicol and then verified by restriction analysis (Table S1). The successfully modified constructs were then transformed into *M. xanthus* DK1622, and the production profile of positive recombinants was analyzed by HPLC-MS (Figure S6-I).

The mutant strains no longer produced disorazols. The missing peaks indicate that all these modules and genes are vital for the disorazol biosynthetic pathway. Without any one of these genes, no disorazols are produced. It is, however, still possible that the significant changes in the cluster architecture that were caused by the gene inactivation may have affected the expression of the biosynthetic enzymes (Figure S6-II).

Biological evaluation

Cell lines were obtained from the German Collection of Microorganisms and Cell Cultures (*Deutsche Sammlung für Mikroorganismen und Zellkulturen*, DSMZ) or were part of our internal collection and were cultured under conditions recommended by the depositor. Half-inhibitory concentrations (IC_{50}) in terms of growth inhibition were determined as described previously[1]. In brief, cells were treated in 96-well plates with serial dilutions of disorazol A_1 and A_2 for 5 d. Cell viability was assessed via tetrazolim salt reduction and average IC_{50} values were obtained in two independent experiments by sigmoidal curve fitting.

Supplemental references

1. Herrmann, J., Hüttel, S., & Müller R. Discovery and biological activity of new chondramides from Chondromyces sp. *ChemBioChem*. **14**, 1573-1580 (2013).
